# Dynamic patterns of gene expression and regulatory variation in the maize seed coat

**DOI:** 10.1186/s12870-023-04078-1

**Published:** 2023-02-07

**Authors:** Juan Li, Liangfa Wang, Jiong Wan, Kuntai Dang, Yuan Lin, Shujun Meng, Xiaoqian Qiu, Qiyue Wang, Jiawen Zhao, Liqin Mu, Hongbing Luo, Dong Ding, Zehui Chen, Jihua Tang

**Affiliations:** 1 College of Agronomy, Hunan Agricultural University, Changsha, 410128 China; 2 National Key Laboratory of Wheat and Maize Crop Science; Collaborative Innovation Center of Henan Grain Crops, College of Agronomy, Henan Agricultural University, Zhengzhou, 450002 China; 3 Institute of Crop Germplasm Resources, Guizhou Academy of Agricultural Sciences, Guiyang, 550006 China; 4 Hebi Academy of Agricultural Sciences, Hebi, 458030 China; 5 Institute of Upland Food Crops, Guizhou Academy of Agricultural Sciences, Guiyang, 550006 China; 6 The Shennong Laboratory, Zhengzhou, 450002 China

**Keywords:** Maize, Seed coat, Heterosis, RNA-seq

## Abstract

**Background:**

Seed size is an important factor contributing to maize yield, but its molecular mechanism remains unclear. The seed coat, which serves as one of the three components of the maize grain, determines seed size to a certain extent. The seed coat also shares the maternal genotype and is an ideal material for studying heterosis.

**Results:**

In this study, the self-pollinated seeds of the maize hybrid Yudan888 and its parental lines were continuously collected from 0 day after pollination (DAP) to 15 DAP for phenotyping, cytological observation and RNA-seq. The phenotypic data showed that 3 DAP and 8 DAP are the best time points to study maize seed coat heterosis. Cytological observations indicated that maize seed coat heterosis might be the result of the coordination between cell number and cell size. Furthermore, the RNA-seq results showed that the nonadditive genes changed significantly between 3 and 8 DAP. However, the number of genes expressed additively was not significantly different. Our findings suggest that seed coat heterosis in hybrid is the result of nonadditive expression caused by dynamic changes in genes at different time points during seed expansion and seed coat development. Gene Ontology (GO) enrichment and Kyoto Encyclopedia of Genes and Genomes (KEGG) pathway enrichment indicated that genes related to DNA replication, cell cycle regulation, circadian rhythms and metabolite accumulation contributed significantly to hybrid seed coat heterosis.

**Conclusion:**

Maize seed coat phenotyping allowed us to infer that 3 DAP and 8 DAP are important time points in the study of seed coat heterosis. Our findings provide evidence for genes involved in DNA replication, cell cycle regulation, circadian rhythms and metabolite accumulation in hybrid with high or low parental expression as major contributors to hybrid seed coat heterosis.

**Supplementary Information:**

The online version contains supplementary material available at 10.1186/s12870-023-04078-1.

## Background

Heterosis has been widely used in plant breeding [1]. Due to heterosis, hybrid F_1_ generations are superior to both parents in terms of viability, plant height, yield, stress and disease resistance [2]. It is hypothesized that the difference in gene expression between a hybrid and its parents is one of the main causes of heterosis [3, 4]. Genetic models to explain the increased yield of hybrids consider the interaction of alleles at many loci generating altered expression levels and patterns [5, 6]. Whole-genome transcriptomes have shown that in developing rice leaves and panicles, differentially expressed genes (DEGs) between hybrids and their parents were overrepresented during energy metabolism and transport [7]. Changes in epigenetic states such as DNA methylation, small RNA production and histone modification have been found in hybrid genomes [8, 9]. Genetic mapping has been used to identify the detailed genetic loci contributing to hybrid performances and investigate their mechanisms, in a number of plant species including Arabidopsis, rice, maize, sorghum, and tomato [10–14]. Rice GAI homologues ectopically expressed in the F_1_ generation of *Arabidopsis thaliana* caused significant plant height changes [10]. The major quantitative trait gene *Ghd7*, which encodes a CCT domain protein in rice, was identified as a key factor for improving the yield and adaptability of the excellent hybrid Shanyou63 and other indica rice varieties [11]. The orthologue of the flowering locus *SFT* for tomato [12] and *Hd3a* for rice [13], has been shown to have single-gene overdominance in yield traits. However, there have been many studies on heterosis, and the genetic mechanism and molecular basis of heterosis remain unclear.

Seed size is an important component of grain yield and a key trait for crop domestication [15]. Seed size is coordinately determined by the development of the triploid endosperm, diploid embryo, and seed coat [7, 16]. The seed coat which is developed from the integument and shares the maternal genotype protects the embryo and endosperm and grows coordinately with them [17]. It plays an important role in the seed filling process [18]. Therefore, the seed coat determines the yield of cereal crops to a certain extent. The development of the seed coat and endosperm takes precedence over that of the embryo. In the early stage of seed development, from double fertilization to 6 days after pollination (DAP), the growth of the seed coat is accompanied by enlargement of the endosperm. Within 72 h after fertilization, the endosperm converts to the multinuclear stage [19]. At 3–6 DAP, the endosperm of maize is in the cellularization stage, during which the cytoskeleton recognizes the nuclei and forms the microtubule system between the nuclei. All nuclei are separated and cytoplasmic division is carried out at the same time to form normal cells [20]. Eight to 12 DAP is the endosperm cell differentiation stage, during which nutrients and storage materials accumulate alongside cell proliferation and enlargement [21]. The morphological changes of the seed coat occur only when the endosperm begins to develop, and the development of the embryo does not promote the growth and differentiation of the seed coat [22, 23]. Thus, the seed coat coincides with the enlargement of the seed in the early stage of development. In addition, the seed coat regulates the accumulation of phloem assimilates in seeds during seed development [24]. At the beginning of the filling stage at 12 DAP, the seed coat thickness is continuously compressed until the seed coat thickness decreases to only a few layers of cells. At this stage, the seed coat cell wall thickens, providing physical protection for future seed dormancy. The function of the seed coat during development may be to use its own starch for endosperm expansion and to provide more space for endosperm development.

An impressive array of genomic and regulatory variations in kernel traits has been observed between maize inbred lines. However, how these variations affect gene expression in hybrids remains unknown. Since the integument does not participate in the fertilization process, the seed coat differentiates primarily from the integument is determined by the maternal plant and shares the maternal genotype [25]. Therefore, the genotype of the seed coat of the F_1_ hybrid and its parents is still the same as that of the maternal plant after fertilization. Because seed size is a heterotic trait, the seed coat of F2 seeds obtained by self-pollination of F_1_ plants shows heterozygosity, which is a good model for studying heterosis. However, the genetic basis of heterosis in the seed coat has remained largely uncharacterized. To this end, this study attempted to better define gene expression variations between seed coats of maize inbred lines, how these variations affect gene expression levels in F_1_ hybrids, and the relative contributions of cis- and trans-variantsto seed coat heterosis. In this study, the self-pollinated kernels of the hybrid Yudan888 and its parental lines were continuously collected from 0 to 15 DAP for phenotyping, cytological observation and RNA-seq. The phenotypic data showed that 3 DAP is an important time point for the expansion of the seed coat area in the hybrid, and 8 DAP is the key time point when the seed coat area of the hybrid significantly extends in the parents.Three DAP and 8 DAP are the best time points to study maize seed coat heterosis. According cytological observations at 8 DAP, we speculate that the heterosis of the seed coat may be the result of the coordination between cell number and cell size. Furthermore, the RNA-seq results at 3 DAP and 8 DAP indicated that the number of genes expressed nonadditively changed significantly in the hybrid. However, the number of genes expressed additively was not significantly different. Our findings suggest that seed coat heterosis is the result of nonadditive expression caused by dynamic changes in genes at different time points. These dynamic change suggests that a dose effect of gene expression results in heterosis. GO enrichment and KEGG analysis indicated that at 3 DAP, the early stage of seed coat development, genes related to DNA replication and cell cycle regulation contributed significantly to heterosis. At 8 DAP, the hybrid weakened the cell cycle by enhancing metabolite accumulation combined with increased kernel weight and stabilizing heterosis. We expect that this transcriptome comparison will provide a new step towards understanding the causative mechanisms of altered gene expression in hybrids and the molecular mechanisms of maize seed coat heterosis.

## Results

### The seed coat area of hybrid and its parents was significantly different at 8 DAP

**Fig. 1:**
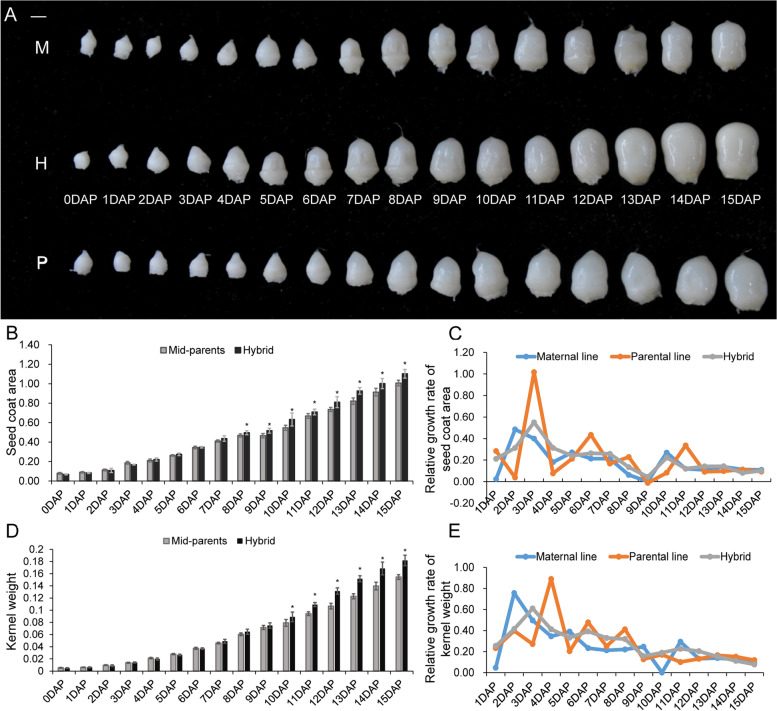
Comparisons of phenotypic variations in the hybrid and its parental lines during seed coat development. **A** Image of seeds from the hybrid (H) and its parental lines (M for maternal line and P for paternal line) at 0–15 DAP. Scale bar, 2 mm. **B** The variation in seed coat area at 0–15 DAP of the hybrid and the mid-parent value. **C** The relative growth rate of seed coat area in the hybrid and its parental lines on 0–15 DAP. **D** The variation in kernel weight during 0–15 DAP of the hybrid and the mid-parent value. **E** The relative growth rate of kernel weight in the hybrid and its parental lines at 0–15 DAP

The seed coat area and kernel weight from 0 to 15 DAP in the hybrid and parental lines were measured continuously (Fig. 1A). At 0 DAP, the seed coat area of the hybrid was lower than that of both the mid-parental value (MPV) and the maternal line. At 6 DAP, the hybrid values began to exceed the MPV but were still lower than those of the maternal line. With the expansion of the seed coat, the hybrid value continued to be higher than the MPV at 7 DAP and slightly exceeded the value of the maternal line. At 8 DAP, there was a significant difference (P value = 0.034) between the hybrid and the MPV regarding seed coat area (Fig. 1B). From 9 to 15 DAP, the seed coat area of the hybrid was increasingly different from the MPV and paternal line values (Table S1). In conclusion, 8 DAP is the inflection point and the entry point of heterosis in the seed coat of maize. The kernel weight of the hybrid was significantly different from that of the MPV after 10 DAP (Fig. 1D). These results indicated that the heterosis of the seed coat area occurred prior to changes in seed weight.

In the seed coat area, the relative growth rate of the hybrid and paternal line peaked at 3 DAP, and the maternal line grew most rapidly at 2 DAP (Fig. 1C). In terms of the relative increasing rate of kernel weight, the value of the hybrid was the highest at 3 DAP, while it was highest at 2 DAP for the maternal line and at 4 DAP for the paternal line (Fig. 1E). In general, seed coat area and kernel weight showed a large increase at 2–4 DAP. More importantly, 3 DAP was found to be a critical time point for hybrid seed coat development.

### Relative cell numbers in the seed coat

**Fig. 2:**
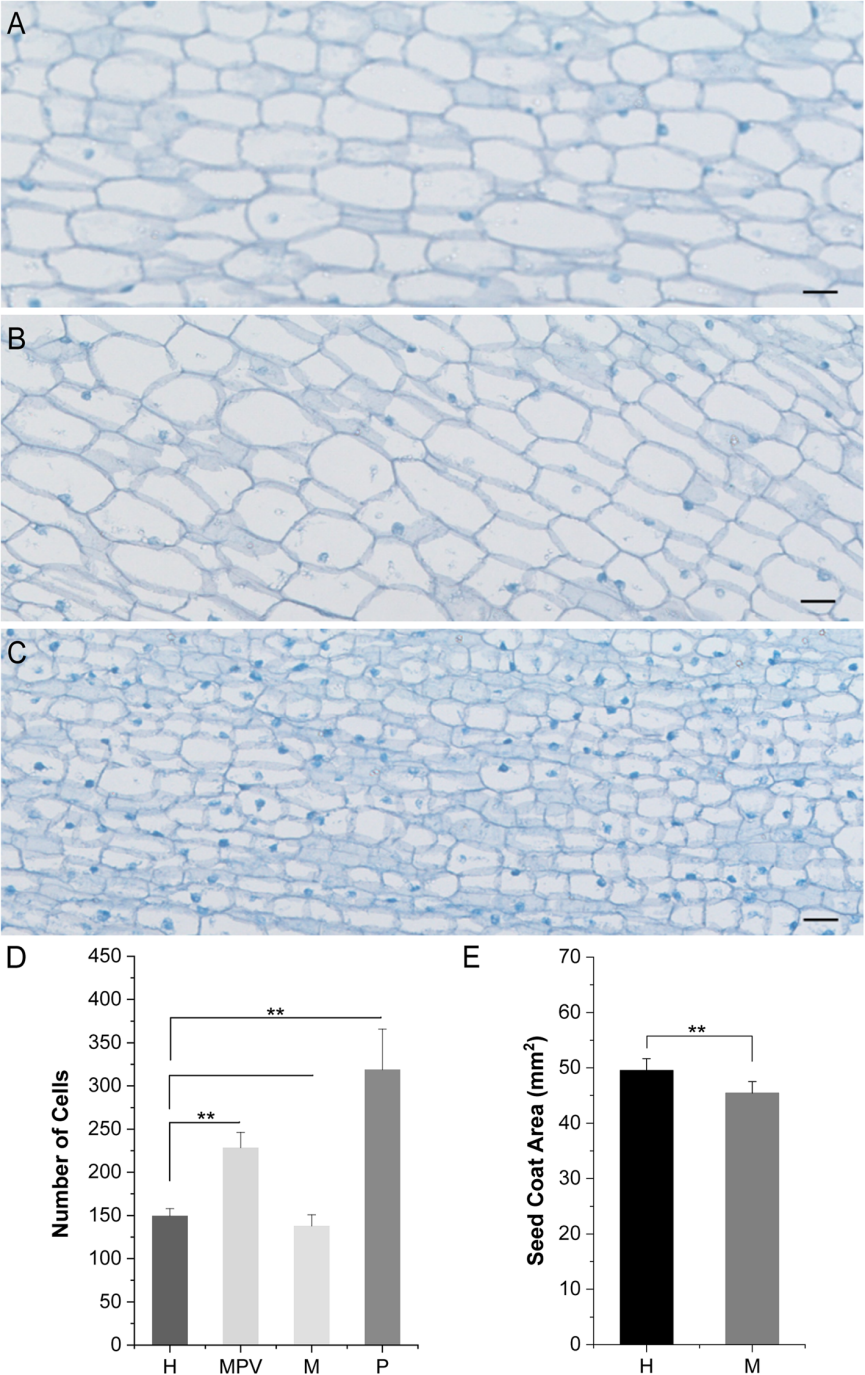
Seed coat cell number per unit area at 8 DAP in the hybrid and its parental lines. **A** Micrographs of the seed coat cell number per unit area in the maternal line. **B** Micrograph of seed coat cell numbers per unit area in the hybrid. **C** Micrograph of seed coat cell numbers per unit area in the paternal line. **D** Comparison of the number of seed coat cells per unit area between the hybrid (H) and maternal line (M), paternal line (P) and mid-parent value (MPV). The cell numbers were counted in a 0.1 mm.^2^ area, with 20 um scale bars. **E** Comparison of seed coat area between the hybrid (H) and maternal line (M)

To understand whether the increased seed coat area in the hybrid was due to changes in cell number or cell size, we counted the number of cells in the seed coat per unit area (0.1 mm^2^) (Fig. 2). The middle longitudinal section of seed embryos collected at 8 DAP was obtained (Fig. 2A-C). The number of cells per unit area for the paternal line (319 cells per 0.1 mm^2^) was higher than that for the hybrid (149.7 cells per 0.1 mm^2^), the maternal line (138 cells per 0.1 mm^2^) and the MPV (228.5 cells per 0.1 mm^2^) at 8 DAP (Fig. 2D). Although there was no difference in the number of cells per unit area between the hybrid and maternal line at 8 DAP, there was a significant difference in seed coat surface area (Fig. 2D, E). Thus, compared with the maternal line, the cell number of the hybrid was determined by its larger seed coat area. The differences in cell number and seed coat area between the hybrid and MPV were significant (Fig. 2D), suggesting that the difference in seed coat size between the hybrid and parents might be the result of the coordination between cell number and cell size.

### Analysis of expressed gene in seed coat

To investigate the expression of genes before and after the time points at which the seed coat of the hybrid line and its parents showed significant differences, we performed RNA-seq on 18 samples at 6 time points (2–4 DAP and 8–10 DAP) from the hybrid and parental lines, with 3 biological replicates for each sample. In total, 54 libraries and 2.4 billion high-quality reads were generated. The reads were then mapped to the maize B73 reference genome (RefGen_V4 [26]) by Hisat [27]. Approximately (87%) of reads were uniquely mapped (Table S2) and used to calculate the normalized gene expression level as fragments per kb of transcript per million mapped reads (FPKM). There was a high correlation among the three biological replicates at each time point (average R^2^ = 0.93) (Table S3). All the above results indicated that the sampling quality of this survey was high, and that the RNA-seq sequencing data were accurate and reliable. Thus, we took the average FPKM value of the three replicates as the expression level for the sample at each time point. The Principal Component Analysis (PCA) revealed that the 18 samples at six time points from three genotypes were assigned to two stages, and that 2–4 DAP were clearly distinguished from 8–10 DAP (Fig. S1). Moreover, the RNA-seq data of 2–4 DAP were overlapping (Fig. S1).

**Fig. 3:**
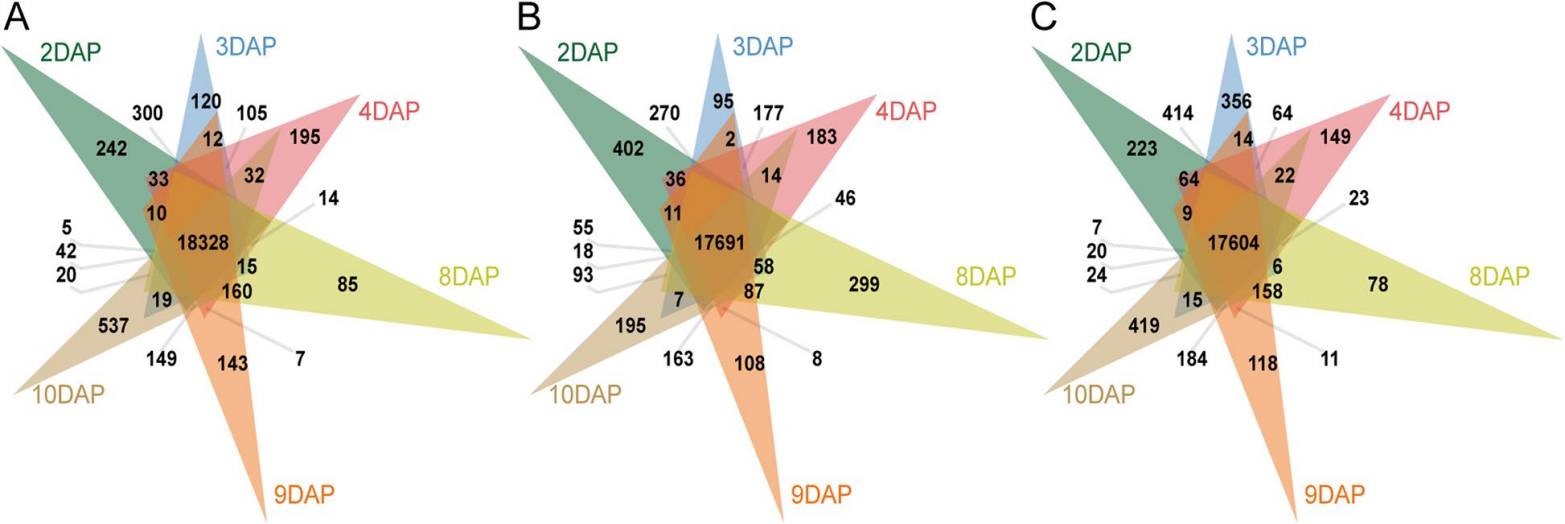
Venn diagram analyses of expressed genes at 6 time points in the hybrid and its parental lines. **A** hybrid Yudan888. **B** maternal line. **C** paternal line

There were 39,005 annotated genes in the B73 AGP_v4 genome [26]. The transcriptomes of both the hybrid and parents exhibited very similar distributions in the number of expressed genes at each time point. In detail, the total numbers of genes expressed by the hybrid, maternal and paternal lines at six time points were 23,516, 23,596 and 23,247, respectively. A total of 17,691, 18,328 and 17,604 genes overlapped at these 6 time points, accounting for 75.23%, 77.67% and 75.73% of the total expressed genes (Fig. 3A-C), respectively. Taken together, the results of overlapping expressed genes in the hybrid and its parental lines showed that the growth and development of the seed coat was mainly due to gene expression level rather than the change in gene expression.

### Differentially expressed gene identification

**Fig. 4:**
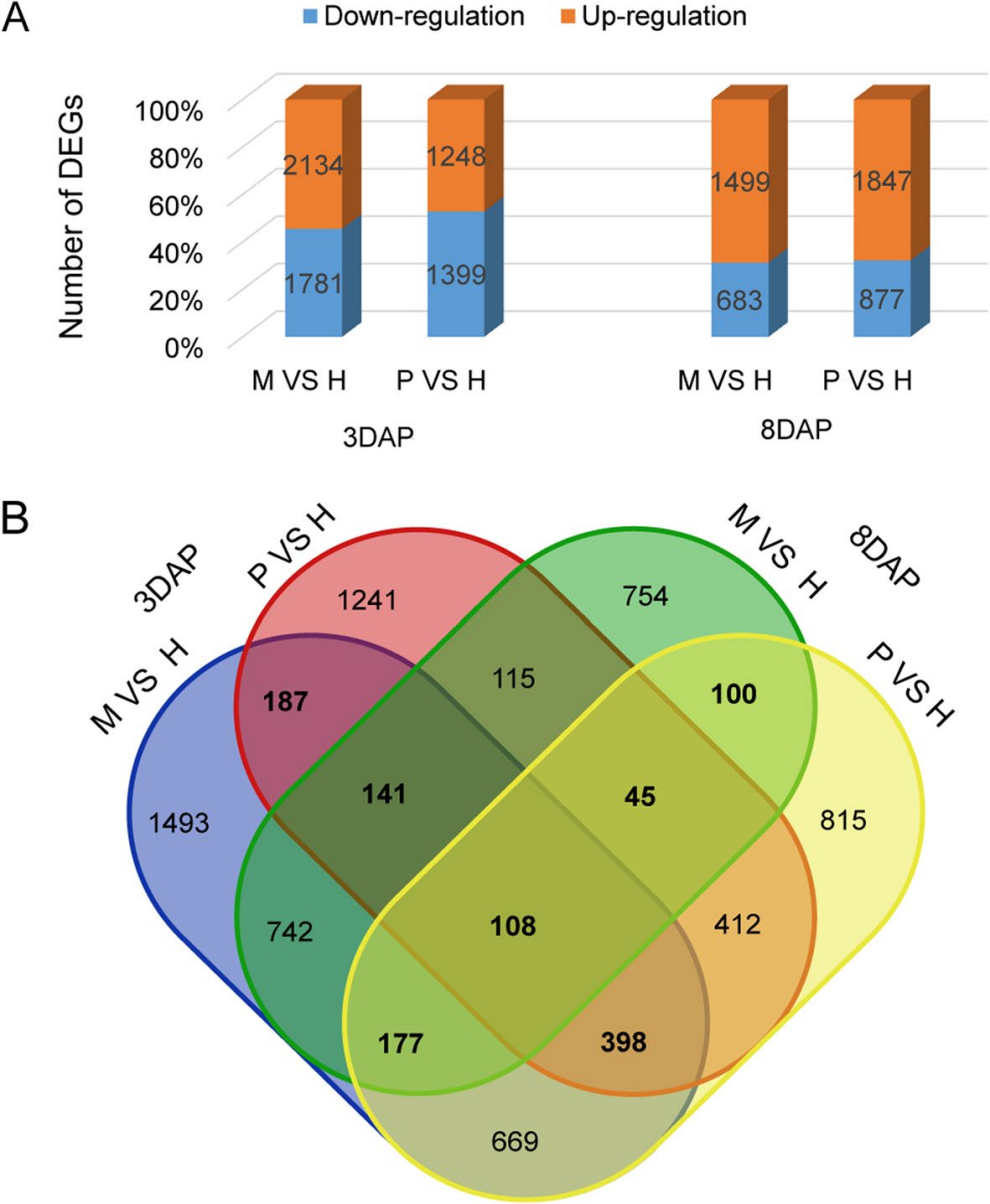
Identification of differentially expressed genes (DEGs) between the hybrid and parents on 3 DAP and 8 DAP. **A** Statistics of up- or downregulated genes between the hybrid and parents are shown by coloured columns at 3 DAP and 8 DAP. **B** Total numbers of DEGs in the maternal line (M) vs. hybrid (H) and paternal line (P) vs. hybrid(H) on 3 DAP, maternal line (M) vs. hybrid (H) and paternal line (P) vs. hybrid (H) on 8 DAP by Venn diagram analysis. Bold numerals indicate commonly expressed DEGs between the hybrid and parents

It was suggested that the hybrid showed significant differences from its parents at 8 DAP, while the relative growth rate of the hybrid seed coat area and kernel weight peaked at 3 DAP. Therefore, we focused on RNA-seq data at 3 DAP and 8 DAP for subsequent analysis. In DEG analysis, the gene expression levels in the hybrid were compared one-by-one with its parental lines, with a difference in the fold change (FC) ≥ 2 and (FC) ≤ -2 denoted as upregulated expression and downregulated expression, respectively. Some DEGs were highly or weakly expressed in either the hybrid or parental lines. At 3 DAP, a total of 3915 DEGs varied between the hybrid and maternal line (2134 (54.50%) upregulated and 1781 (45.50%) downregulated), and 2647 DEGs varied between the hybrid and paternal line (1248 (47.15%) upregulated and 1399 (52.85%) downregulated). Compared with that at 3 DAP, the proportion of DEGs upregulated at 8 DAP increased sharply. A total of 2182 DEGs varied between the hybrid and maternal line (1499 (68.70%) upregulated and 683 (31.30%) downregulated), and 2724 DEGs varied between the hybrid and paternal line (1847 (67.80%) upregulated and 877 (32.20%) downregulated) (Fig. 4A). The differences in the upregulation and/or downregulation of gene expression between the hybrid and its parents varied across comparisons at 3 DAP and 8 DAP. Some DEGs had genotype-specific expression and were thus identified as genotype-specific unigenes (Fig. 4B). Venn diagram analysis indicated that 1156 genes were DEGs (highlighted as black bold font) between the hybrid and its parents at both time points (Fig. 4B). Out of these 1156 genes, 834 DEGs (sum of bold font numerals) were commonly observed between M vs. H and P vs. H at 3 DAP. However, 430 DEGs (sum of bold font numerals) were commonly observed between M vs. H and P vs. H at 8 DAP (Fig. 4B). In the hybrids at both time points, 108 DEGs were found to be commonly differentially expressed (Fig. 4B).

### Expression patterns of DEGs

To explore and categorize the trends for changes in expression, DEGs in 3 DAP and 8 DAP samples were divided into 13 possible hybrid and parental expression patterns (Table 1). Gene expression patterns were divided into additive expression and nonadditive expression. We found no significant changes in the number of additively expressed DEGs between the two time points. The expression pattern of most genes in the hybrid at the two time points was additive (85.18% at 3 DAP and 86.45% at 8 DAP). However, the effects of nonadditive DEGs were quite different, especially the dominantly expressed DEGs (classes 5, 7, 10 and 12). That is, the gene expression in the hybrid was similar to that of one parent, which accounted for more than 13% of all DEGs at both time points. At 3 DAP, the number of dominantly expressed genes with higher paternal expression accounted for the highest proportion in the nonadditive classes, while at 8 DAP, the number of dominantly expressed genes with higher maternal expression was the most abundant. The number of overdominantly expressed genes with transitional downregulation (0.35% at 3 DAP and 0.14% at 8 DAP) was relatively higher than that with transitional upregulation (0.10% at 3 DAP and 0.08% at 8 DAP).

**Table 1:** Classification of expression patterns of DEGs in hybrids and their parental lines at 3 DAP and 8 DAP

Categories	Expression patterns of genes														Total DEGs
	Addtivity			Nonaddtivity											
	M vs. P Non-DEGs	M vs. P DEGs		Dominance				Overdominance							
				M vs. P DEGs				M vs. P Non-DEGs		M vs. P DEGs		M vs. P Non-DEGs	M vs. P DEGs		
				ELD_M		ELD_P		Transgressive upregulation				Transgressive downregulation			
Cl	2	6	11	10	5	7	12	1	8		9	3	4	13	
asses															
Relative expression	M-H-P	M-H-P	M-H-P	M-H-P	M-H-P	M-H-P	M-H-P	M-H-P	M-H-P		M-H-P	M-H-P	M-H-P	M-H-P	
3 DAP	17,045	1075	1594	622	790	1385	529	15	3		4	70	12	0	23,145
Sum	19,714			1412		1914		22				82			
Proportion of total DEGs(%)	85.18			6.10		8.27		0.10				0.35			
8 DAP	16,899	1129	1432	1360	403	998	239	12	3		2	29	3	0	22,509
Sum	19,460			1763		1237		17				32			
Proportion of total DEGs(%)	86.45			7.83		5.50		0.08				0.14			

DEG expression patterns were classified according to the expression levels of the parents and hybrid. Additive expression of genes: classes 2, 6 and 11 (blue); dominant expression of genes: classes 10, 5, 7 and 12 (green); and overdominant expression of genes: classes 1, 8, 9, 3, 4 and 13 (red). Classes 1, 8 and 9 represent transgressive upregulation and classes 3, 4 and 13 represent transgressive downregulation [28]. Diagrams of each class represent the relative expression levels observed in the maternal line (left), hybrids (middle), and paternal line (right). DEGs, differentially expressed genes, M, maternal line, H, hybrid, P, paternal line, ELD, expression–level dominance, additivity: H≈ 1/2 (M + P), nonadditivity: H > 1/2 (M + P) or H < 1/2 (M + P), ELD_M: H ≈ M > P or H ≈ M < P, ELD_P: H≈ *P > *M or H ≈ P < M, transgressive upregulation: H > M and H > P, transgressive downregulation: H < M and H < P

We summarized 13 possible hybrid and parental expression patterns into mid-parent, high-parent and low-parent categories to analyse the expression changes at both time points. By comparing the number of DEGs in three expression patterns at both time points, it was found that the number of low-parent DEGs changed the most, followed by the number of high-parent DEGs, while the number of mid-parents DEGs changed the least (Table S4). According to the analysis of the fold changes at both time points, it was suggested that the change in the number of low-parent and high-parent DEGs may play a role in hybrid seed coat heterosis.

### Functional classification by Gene Ontology (GO) and Kyoto Encyclopedia of Genes and Genomes (KEGG) pathway enrichment analysis

**Fig. 5:**
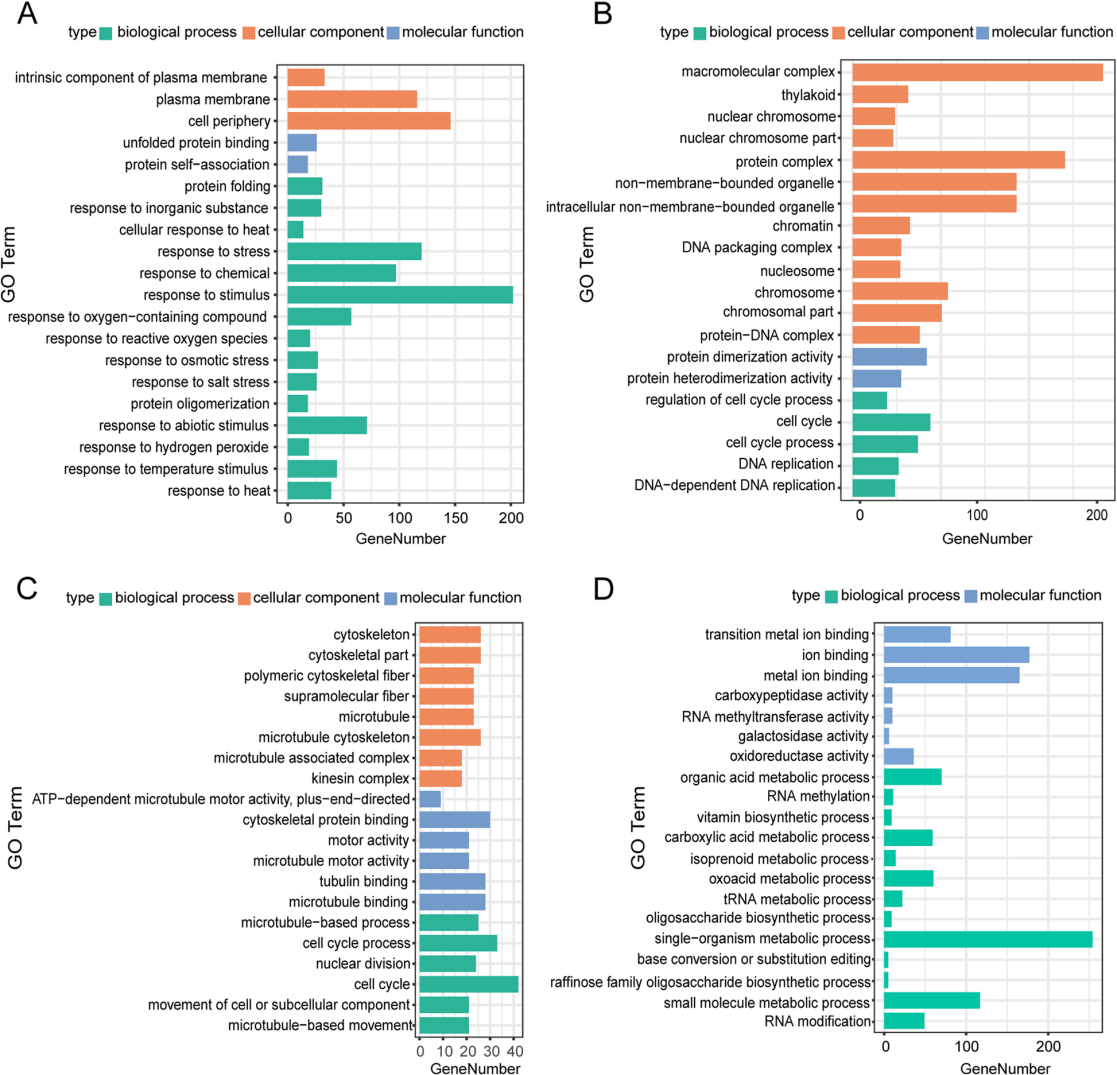
GO enrichment analysis of genes with low- parental expression and high -parental expression at 3 DAP and 8 DAP. **A** The top 20 GO terms of the shared genes with low- parental expression at 3 DAP. **B** The top 20 GO terms of the shared genes with high- parental expression of 3 DAP. **C** The top 20 GO terms of the shared genes with low- parental expression at 8 DAP. **D** The top 20 GO terms of the shared genes with high- parental expression at 8 DAP

The DEGs with high and low parental expression at 3 DAP and 8 DAP were annotated by GO. The enrichment of GO terms with low-parental expression at 3 DAP showed that a large number of DEG categories related to stress were significantly enriched in biological processes, followed by cellular components, and categories related to the plasma membrane (Fig. 5A). The highly expressed DEG GO enrichment results at 3 DAP suggested that categories related to chromosome assembly and processing were enriched in cellular components, and categories related to the cell cycle and DNA replication were enriched in biological processes (Fig. 5B). The GO enrichment results for low-parental expression at 8 DAP demonstrated that the categories related to cytoskeleton and microtubule were enriched in cellular component, and the categories enriched in biological process were mainly related to cell cycle, nuclear division and microtubule (Fig. 5C). The GO enrichment results for highly expressed DEGs at 8 DAP indicated that categories related to metabolism were enriched (Fig. 5D).

**Fig. 6:**
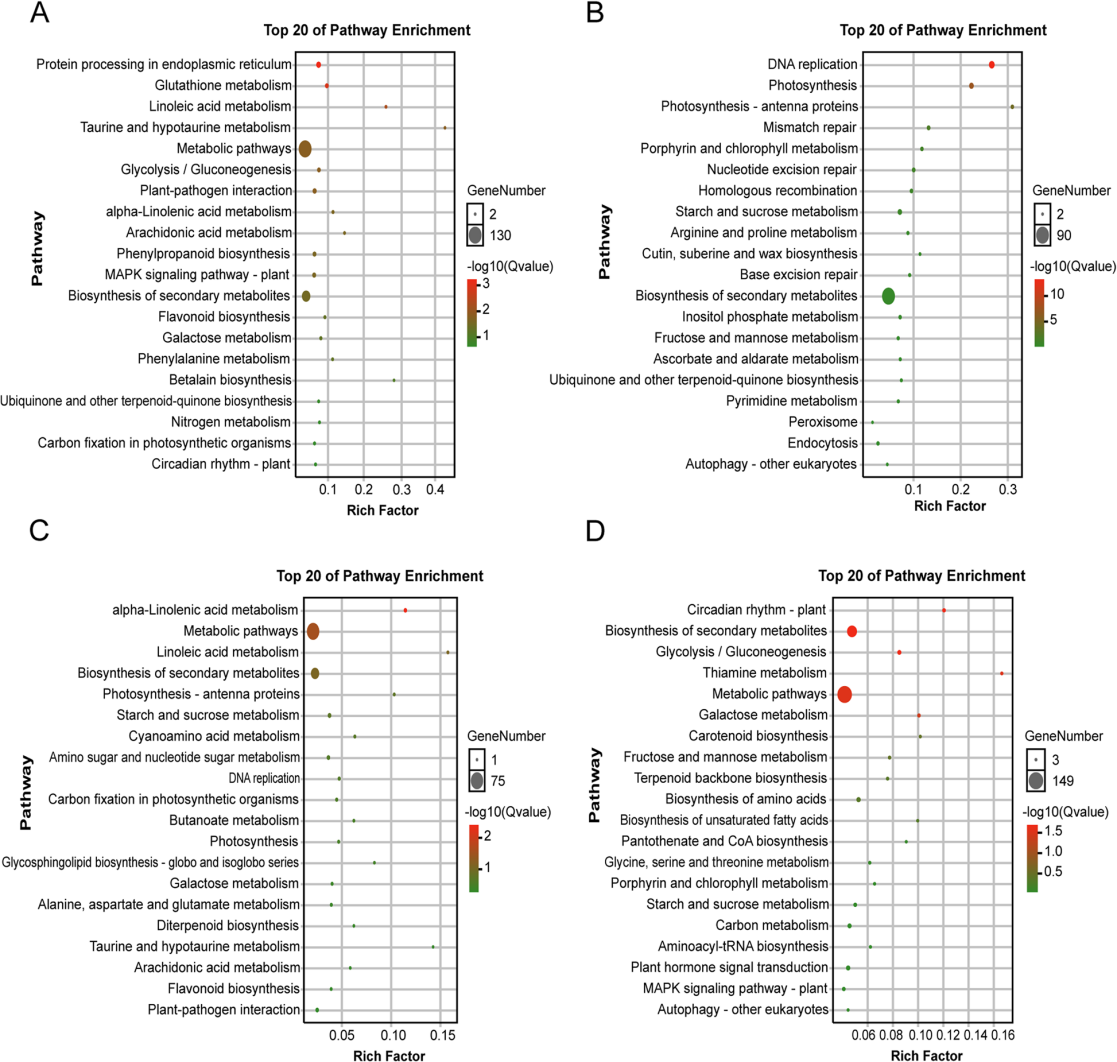
KEGG analysis of genes with low- parental expression and high- parental expression at 3 DAP and 8 DAP. **A** The top 20 KEGG pathways of the shared genes with low- parental expression at 3 DAP. **B** The top 20 KEGG pathways of the shared genes with high- parental expression at 3 DAP. **C** The top 20 KEGG pathways of the shared genes with low- parental expression at 8DAP. **D** The top 20 KEGG pathways of the shared genes with high-parental expression at 8 DAP

The KEGG pathway enrichment analysis was carried out to further understand the biological function of genes and their interactions. We performed KEGG enrichment analysis on DEGs with high and low-parental expression at 3 DAP and 8 DAP, respectively (Fig. 6). Among the 3 DAP low-parental expressed DEGs, metabolic pathways, biosynthesis of secondary metabolites, protein processing in the endoplasmic reticulum and glutathione metabolism were significantly enriched (Fig. 6A). For the 3 DAP high-parental expressed DEGs, the biosynthesis of secondary metals, DNA replication and photosynthesis were significantly enriched (Fig. 6B). Metabolic pathways, the biosynthesis of secondary metabolites and alpha linolenic acid metabolism were significantly enriched in 8 DAP low-parental expressed DEGs (Fig. 6C). In contrast to DEGs with high parental expression at 3 DAP, DNA replication was also enriched in the low-parental expression DEGs at 8 DAP. For the high-parental expression DEGs at 8 DAP, circadian rhythms, the biosynthesis of secondary metals, glycolysis/gluconeogenesis, thiamine metabolism, metabolic pathways, galactose metabolism and other pathways were significantly enriched (Fig. 6D).

### Weighted gene co-expression network analysis (WGCNA)

**Fig. 7:**
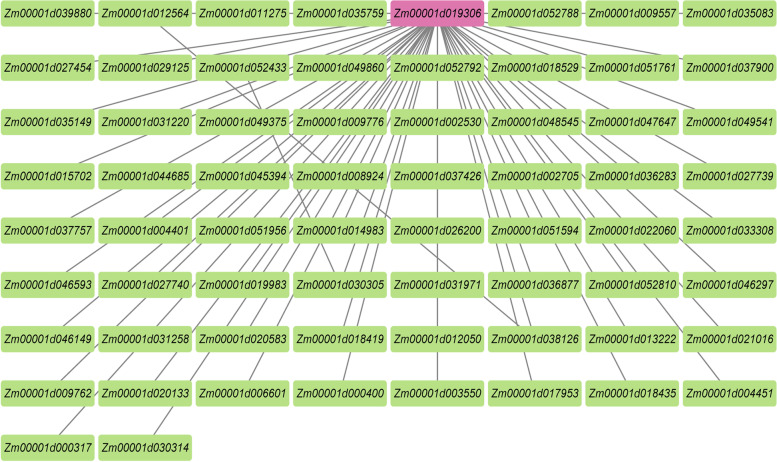
The gene network of *Zm00001d019306*

To identify the specific genes highly related to seed coat area, we constructed co-expression networks by using the transcriptome data from comparisons of the hybrid and its parents at 6 time points, and associated the co-expression module with seed coat area traits. The results showed that these genes should be divided into 41 modules (Fig. S2). By observing the correlation between modules and samples, it was found that the absolute value of the correlation coefficient between the MEyellow (0.86) and MEsienna3 (0.82) modules was the highest (Fig. S2). In other words, these two modules have the highest correlation with seed coat area. In these two modules, the genes with the top 100 weight values were screened to construct the regulation network diagram (Fig. 7, Fig. S3). First, the module MEyellow revealed that *Zm00001d019306* was a hub gene (Fig. 7). However, in module MEsienna3, the regulatory network was not as simple and clear as that in MEyellow. We chose genes with a degree value of more than 10 as hub genes. Therefore, the hub genes in module MEsienna3 were *Zm00001d006330*, *Zm00001d043289*, *Zm00001d007181*, *Zm00001d043290*, *Zm00001d033510*, *Zm00001d024732*, *Zm00001d015025*, *Zm00001d006331* and *Zm00001d047796* (Fig. S3, Table S5).

### Validation of DEGs by quantitative real-time PCR

**Fig. 8:**
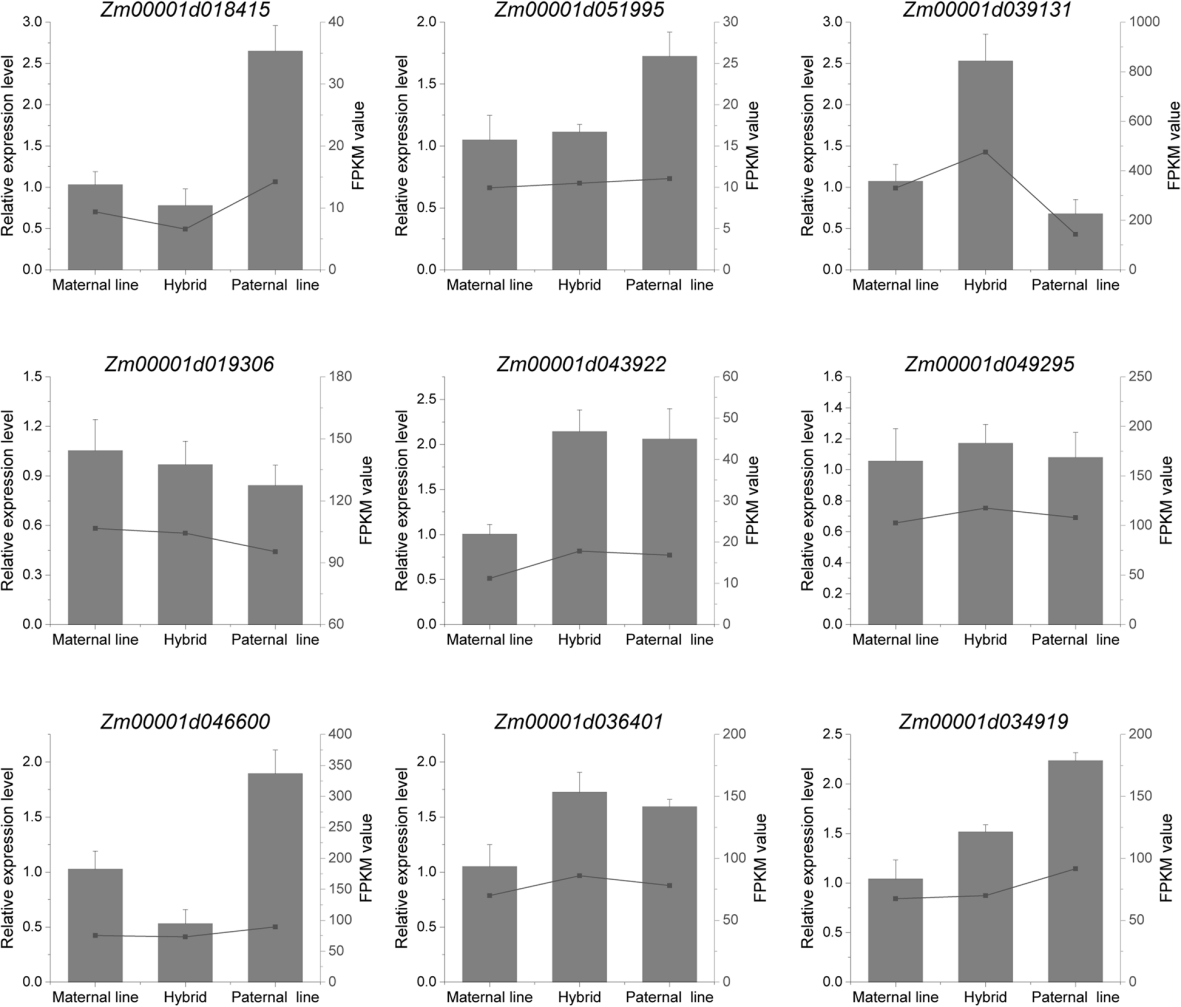
The relative expression levels of 9 differentially expressed genes at 8 DAP as assessed by qRT‒PCR. The qRT‒PCR relative expression levels are shown in the column diagram in the figures, and the FPKM value obtained by RNA-seq is represented by the lines in the figure

The expression patterns of nine DEGs were further confirmed by quantitative real-time PCR (qRT‒PCR). We compared the transcriptional profiles of each gene with the seed coat RNA samples from the hybrid and its parents at different time points. The data confirmed that the expression patterns of all 9 DEGs were consistent with the expression levels obtained by RNA-seq analysis (Fig. 8, Fig. S4).

## Discussion

Heterosis has been known for centuries [29]. Maize seeds are characterized as having heterosis, and seed size is determined by the maize seed coat area to a certain extent. The maize seed coat develops from the ovary wall and carries a maternal genotype. Due to its genetic stability and fixed heterozygosity, the seed coat of F2 seeds obtained by self-pollination of F_1_ plants carries a heterozygous genotype, which makes it a good model for studying heterosis. Therefore, exploring the seed coat area of seeds obtained by self-pollination of maize F_1_ plants and their self-crossed parents can deepen our understanding of maize seed coat heterosis, and in turn seed size and yield heterosis.

The initiation of seed coat development depends on the fusion of the central cell with one of the sperm cells [30]. In our study, the relative growth rate of the seed coat area of the hybrid and paternal line peaked at 3 DAP, while the maternal line grew most rapidly with the peak at 2 DAP. This phenomenon may result from the fact that the synthesis of auxin production in the endosperm after fertilization removes the PRC2 block on seed coat development, to promote the coordinated development of the seed coat and endosperm [31]. There was a significant difference between the hybrid and mid-parental value for seed coat area at 8 DAP, suggesting that heterosis of the seed coat area might be the result of the coordination between cell number and cell size (Fig. 2). These results were consistent with the GO enrichment results from the transcriptome comparison between the hybrid and parents in the early stage of seed coat growth and development, suggesting that heterosis of the seed coat area resulted from the enhanced cell cycle and cell division activity in the early stage. Previous studies have reported that plant growth heterosis basically results from changes in cell number, similar to most plant characteristics [32]. However, the elongation of seed coat cells also affects seed size in Arabidopsis [33]. Our results showed that the maize seed coat developmental program of the hybrid was not dramatically altered, instead, cell proliferation and cell elongation changed.

Transcriptome analysis is a well-established method to identify differential expression levels and regulatory mechanisms of genes from different samples at the transcriptional level [34]. Previous studies in plants have identified heterotic genes associated with many traits between hybrids and parents and have proved that gene dose effects play a role in heterosis [35–37]. RNA-seq was performed on the seed coats of the 2–4 DAP and 8–10 DAP hybrid and parental lines. The expression results indicated that the heterosis of seed coat extension was mainly due to the dosage effect of gene expression rather than none-or-all expression.

The differential expression pattern of DEGs between the hybrid and its parents plays a significant role in heterosis [8, 38]. In hybrids, where 2 different alleles of a gene are combined, the allelic allelic expression may deviate from that of either parent or mid-parent predictions [39]. Concerning the relative gene expression level within a hybrid–parent triad, there are usually two scenarios about the relative gene expression level between the triples of hybrid parents. In the first case, the gene expression of the hybrid showed a cumulative pattern, which was contributed by each allele of parents. This pattern was additive and mainly regulated by cis action. In another case, the expression deviates from the mid-parental level, and other trans acting factors may lead to the change in expression for the corresponding allele in the hybrid [39]. In the present study, the majority (85.18% at 3 DAP and 86.45% at 8 DAP) of genes in the hybrid were additively expressed, suggesting that cis-acting elements played a major role in the regulation of gene expression. The nonadditive genes expressed throughout our entire dataset accounted for less than 15% at each time point but accounted for 54.35–56.24% of the DEGs. This result indicates that the difference in gene expression between the hybrid and parental lines was mainly due to nonadditive effects. Analysis of the expression of nonadditive genes indicated that the allelic expression pattern of the hybrid may not be a simple combination of alleles from both parents, but may be regulated by other genes or trans-action factors. By comparing the expression patterns of hybrids in 3 DAP and 8 DAP, the number and type of additive expressed genes changed slightly (19,714 or 85.18% at 3 DAP to 19,460 or 86.45% at 8 DAP DEGs), but the number and type of highly-expressed and weakly-expressed genes at 3 DAP and 8 DAP varied greatly (Table S4, Fig. 5). In conclusion, it was suggested that the expression of nonadditive genes in hybrids may be the main cause of heterosis in hybrid seed coats.

Three DAP and 8 DAP are the key stages of maize endosperm cellularization and differentiation, respectively [20, 21]. In this study, the results of GO enrichment analysis and KEGG pathway analysis of different expression patterns for the seed coat in hybrid and parental lines at 3 DAP and 8 DAP matched the endosperm development process. In other words, at 3 DAP, compared with the parents, the hybrid increased its seed coat size mainly by enhancing cell cycle activity, weakening the response to stress and codeveloping with the endosperm (Fig. 5A-B). However, at 8 DAP, the hybrid seed coat showed a reduced rate of cell division, instead by strengthening the metabolic process. At this stage, the seed coat changed mainly in coordination with the endosperm to enhance the synthesis and accumulation of nutrients in the seed, to enrich the grain and increase the kernel weight (Fig. 5C-D). This result was supported by the significant difference in kernel weight between the hybrid and MPV at 10 DAP (Fig. 1D). The KEGG analysis results of genes with high parental expression at 8 DAP showed that, pathways related to circadian rhythm and metabolite accumulation were significantly enriched. Changing the circadian rhythm can improve the viability and biomass of hybrids [40]. In addition, evidence of changes in metabolic profiles has been recorded in hybrids [41, 42]. Moreover, seed development is accompanied by metabolic activities for the synthesis and accumulation of stored products, including protein and carbohydrates [43]. Therefore, in addition to genetic control, the final size of seeds may also be affected by metabolic activities [44].

*Zm00001d018415* and *Zm00001d051995*, two genes encoding proliferating cell nuclear antigen(PCNA)protein, showed high parental expression patterns in the GO and KEGG analyses at 3 DAP. PCNA is a key protein in the mitotic DNA replication mechanism of all eukaryotes [45]. It acts as a DNA polymerase δ cofactors and is thus necessary for the synthesis of new DNA strands by forming a homotrimeric complex [29, 46] that binds to various cyclin-CDK complexes [47–49]. In addition, as a sliding clamp, PCNA also plays a role in regulating DNA metabolism, processing DNA damage and controlling the cell cycle [50]. Therefore, the high parental expression of the PCNA gene and its related proteins at 3 DAP may play a role in enhancing DNA replication, repair and metabolism in the early stage of seed coat development, to increase the cell cycle rate and cell number in the hybrid. Moreover, the excessive transcript abundance of these genes in hybrids in the early stage of seed coat development may eventually help to maintain heterosis through dominant gene effects to a great extent. *Zm00001d039131*, encodes ADP-glucose pyrophosphorylase, which catalyses the key steps of the starch synthesis pathway. This gene was enriched in GO terms and KEGG pathways related to metabolism and the biosynthesis of secondary metabolites at 8 DAP when high parental expression was present. This gene has been reported to be associated with seed development and filling and kernel weight [51–53]. Eight to 12 DAP is the stage of endosperm cell differentiation, during which nutrients and storage materials accumulate alongside cell proliferation and enlargement [21]. Seed development is a coordinated process among the seed coat, embryo and endosperm. ADP-glucose pyrophosphorylase was highly parentally expressed at 8 DAP in the hybrid, while the cell cycle-related genes showed a low-parental expression level. This phenomenon may be the result of the rapid development and nutrient filling of the endosperm through enhanced accumulation of metabolites in the hybrid. In other words, during this period, the gene expression pattern of the hybrid changed, that is, cell cycle activity was weakened and metabolic processes were enhanced, to stabilize heterosis by cooperating with the development and filling processes in the endosperm.

WGCNA can be used to analyse the relationship between a gene set and sample phenotype, draw the regulatory network between genes in a gene set and identify key regulatory genes [54]. *Zm00001d019306*, the hub gene of WGCNA in the highest correlation coefficient modules with the highest correlation coefficients, was significantly positively correlated with the seed coat phenotype and encoded, an indole-3-acetyl-leu hydrolase. This enzyme can catalyse the hydrolysis of indole-3-acetyl-L-leucine and release free indole-3-acetic acid (IAA) [55]. Seed development is regulated by phytohormones [44]. Auxin, as a central plant hormone, regulates many biological processes, including the maintenance of meristems, cell division and cell expansion [56–58]. In Arabidopsis, indole-3-acetyl-leu hydrolase regulates the auxin response by regulating auxin homeostasis in the endoplasmic reticulum. Auxin is the key regulator of the plant defence response and the main control factor of plant growth and development. The change in gene activity related to the IAA pathway has potential importance in producing a heterosis phenotype [59, 60]. Previous studies showed that in Arabidopsis, the utilization of the IAA pathway caused differences in the control of auxin on cell proliferation by auxin, resulting in different levels of heterosis [61]. Interestingly, these views are consistent with our results. On the one hand, at 3 DAP, genes related to the defence response in hybrids were significantly downregulated. On the other hand, heterosis of the maize seed coat area is determined by the number of cells. In summary, indole-3-acetyl-leu hydrolase may play a significant role in of the IAA pathway and seed coat heterosis. It may regulate the auxin response by regulating auxin homeostasis in different seed coat development processes, to effectively maintain the balance of hybrid immunity and growth and development regarding resource allocation at different developmental time points of the seed coat.

Finally, the expression levels of 9 genes identified by RNA-seq analysis were verified by qRT‒PCR, and the expression patterns of all tested genes were consistent with the results of RNA-seq, indicating the reliability of RNA-seq data in Yudan888 and its parents (Fig. 8). In conclusion, the genes identified in our study may provide some insights into broader aspects of seed coat heterosis in maize. However, further validation is needed to confirm the association between gene expression patterns and target agronomic traits in maize.

## Materials and methods

### Field experiment and phenotyping

In summer 2020, maize hybrid Yudan888, a widely cultivated maize variety in the Huang-Huai-Hai area of China, together with its parental lines 15S717 and T4691, was planted on the farm of Henan Agricultural University (Yuanyang, China, 113°16’ E, 35°41’ N). The planting density was 75,000 plants per hectare, with 3 replicates each containing 600 seedlings. A randomized block design was adopted in the field experiment. All the plants of the hybrid and parental lines were artificially self-crossed.

Seed coat phenotyping began at 0 DAP and continued daily until 15 DAP, phenotyping was completed between 9 and 11 am each day. The kernel weight was measured immediately after sampling by an analytical balance (ME204E, METTLER TOLEDO) with an accuracy of 0.0001 g. The whole seed coat was peeled off with tweezers and photographed, on a black background. Finally, the seed coat area was calculated by ImageJ software. At least six individual plants per DAP with no less than six kernels per plant were sampled for phenotyping. Univariate ANOVA was performed using IBM SPSS statistics 24 to test the significant differences in the measured the area of the seed coat between the hybrid and parental lines.

Seeds from the centre of three different ears were fixed in FAA buffer (50% ethanol: formaldehyde: acetic acid = 90:5:5 volume) and then vacuumed at 4 °C for further imaging. Kernels from each sample were removed from the FAA solution and rinsed thoroughly in deionized water. Kernels were dissected at the longest part with a scalpel. Each dissected kernel was embedded in paraffin. Sections from kernels ranged from 8 to 10 μm in thickness. The maximum longitudinal cross section of intact kernels was dyed with 1% toluidine blue after dewaxing. Images of the sections were captured with a microscope (Axio Scope A1, Carl Zeiss) fitted with a digital camera (Axiocam 503 colour, Carl Zeiss). ImageJ was used to measure the number of seed coat cells per unit area.

### Sample collection and RNA extraction

RNA-seq samples were taken during the period when hybrid and parental lines showed significant differences in the seed coat areas. The seed coat samples for RNA-seq were collected by manual dissection with a scalpel. Three biological replicates were set for each sample at each time point. Each replicate sample was collected from at least three individual ears. The samples collected were frozen immediately in liquid nitrogen and stored at -80 °C before RNA extraction. Total RNA was extracted from seed coat samples of the hybrid and its parental lines at 2–4 DAP and 8–10 DAP using TRIzol reagent (Invitrogen, United States). Three biological replicates were used for at each DAP. The quality and concentration of each RNA sample were determined using both gel electrophoresis and a NanoDrop 2000 spectrophotometer (Thermo Fisher Scientific, Wilmington, DE). Only RNAs that met the criterion of an OD260/280 ratio of 1.9–2.1 were stored in a − 80 °C freezer for further use.

### RNA-seq and data analysis

A total of 1 μg of RNA was used as the input material for the construction of each RNA library, with the NEBNext® UltraTM RNA Library Prep Kit for Illumina® (NEB, USA). Briefly, mRNA was purified from total RNA by using poly-T oligo-attached magnetic beads. First strand cDNA was synthesized using random hexamer primers and M-MuLV Reverse Transcriptase (with RNase H). After second-strand cDNA synthesis, terminal repair, poly(A) tail addtion sequencing oligonucleotide adaptor ligation, the fragments were purified and subsequently amplified by PCR. The PCR products were assessed with the Agilent Bioanalyzer 2100 system. Finally, the libraries containing 150 bp cDNA inserts in size were generated and sequenced by a commercial service company (Guoke Biotechnology, Beijing, China) on an Illumia Noveseq platform.

All data analyses were based on clean data with high quality [62]. Feature Counts v1.5.0-p3 was used to count the read numbers mapped to each gene (ftp://ftp.ensemblgenomes.org/pub/release-40/plants/fasta/zea_mays/dna/). Multiple testing with the Benjamini‒Hochberg approach for controlling the false discovery rate (FDR) was taken into account by using an adjusted P value. A corrected P value of 0.05 and an absolute fold change of two times were set as the thresholds for significantly differential expression. The normalized gene expression values with FPKM were used for PCA. PCA was performed in R 4.2.1. The GO enrichment analyses were obtained using agriGO v2.0 (http://systemsbiology.cau.edu.cn/agriGOv2/index.php) [63]. The KEGG annotations were obtained on the KEGG website (https://www.kegg.jp/kegg/) [64]. The GO and KEGG enrichment analyses were performed using TBtools [65].

### Classification of differential gene expression patterns

Based on previous studies on gene expression patterns [66–70], the FPKM values of genes were compared between the hybrid and its parents. The expression patterns were divided into additive and nonadditive (dominance or overdominance). A relative expression level of a hybrid gene that was located between parental values was classified as additive (regardless of whether there were DEGs between the parents or not). A relative expression level of DEGs similar to that of the dominant parents was classified as dominant. A relative expression level of DEGs that was higher than that of the parents (excessive upregulation) or lower than that of the parents (excessive downregulation) was classified as overdominance.

### Weighted gene co-expression network analysis (WGCNA)

The assignments of gene co-expression modules using the WGCNA protocol were based on FPKM data (Langfelder and Horvath, 2008). To determine the module related to seed coat surface area, we associated the genes in each module with the seed coat area trait from 2–4 DAP and 8–10 DAP. The genes with an average FPKM > 1 in 54 samples were analyzed. The soft threshold power β for network construction was set to 11. The dynamic tree cutting algorithm with a minimum module size of 50 genes was used for hierarchical cluster cutting; a value of 0.15 was used to merge similar modules. If the p value of the module trait association was less than 0.05, it was defined as a meaningful module. In R 4.0.1, the WGCNA package was used to calculate the correlation weight of each gene and all other genes in the module. The top 100 gene sets in the module were visualized using Cytoscape V3.9.1, and the gene with the highest degree was considered to be a hub gene.

### qRT‒PCR

Nine differentially expressed genes were selected for qRT‒PCR verification, and the first-strand cDNA was synthesized by reverse transcription using total RNA from the seed coat of the hybrid, the maternal line and the paternal line as templates. The primer 3 online toolbox was used to design gene specific primers for qRT‒PCR. The primers used for quantitative PCR are listed in Supplemental Table S6. The reverse transcription and qRT‒PCR reaction system and reaction procedures were in accordance with the instructions for the PrimeScriptTM RT Reagent Kit with gDNA Eraser (TaKaRa) and TB GreenTM Premix Ex TaqTM II (TaKaRa) Kit. Each experiment had 3 biological replicates and 3 technical replicates. The relative expression levels of these genes were analysed by the 2^−ΔΔCt^ method with *Zm-actin-1* serving as the internal reference [71].

## Supplementary Information

**Additional file 1: Fig. S1.** Principal component analysis (PCA) of the RNA-seq data at six time points. Mean fragments per kilobase of transcript per million mapped read (FPKM) values of three biological replicates are used for each genotype at each time point.

**Additional file 2: Fig. S2.** Module-trait relationship of the module eigengene correlation with seed coat area, kernel weight, kernel width, kennel thickness and kernel length using WGCNA.

**Additional file 3: Fig. S3.** Gene networks of hub genes with MEsienna3 module. Top 100 of genes with weight value are shown according their weight value with these hub genes. Genes with a degree value ≥ 10 were marked red.

**Additional file 4:  Fig. S4. **The relative expression levels of 9 differentially expressed genes on 3DAP as assessed by qRT-PCR.

**Additional file 5: Table S1. **Comparison of the seed coat area between the hybrid (H) and the mid-parents value (MPV) from 0 DAP to 15 DAP.

**Additional file 6:  Table S2.** The sequencing data of the samples were compared with the selected reference genomes.

**Additional file 7: Table S3.** The average correlation coefficients of three biological replicates in three marterials at each time point.

**Additional file 8: Table S4. **Statistics of parental expression patterns into high- parent, mid- parent and low- parent at 3 DAP and 8 DAP.

**Additional file 9: Table S5. **Description of hub gene by WGCNA.

**Additional file 10: Table S6.** List of oligonucleotides used for qRT‒PCR.

## Data Availability

The sequence datasets in fastq format of the current study are available in the NCBI Sequence Read Archive (SRA) database under Bioproject PRJNA828527 (https://www.ncbi.nlm.nih.gov/bioproject/PRJNA828527).

## Abbreviations

DAP: Days after pollination
MPV: Mid-parental value
PCA: Principal Component Analysis
DEGs: Differentially expressed genes
GO: Gene Ontology
KEGG: Kyoto Encyclopedia of Genes and Genomes
WGCNA: Weighted gene co-expression network analysis
qRT‒PCR: Real-time quantitative PCR
