# Supplementary material for: Dynamic patterns of gene expression and regulatory variation in the maize seed coat
Source: BMC Plant Biol. 2023 Feb 7;23:82. doi: 10.1186/s12870-023-04078-1 (PMC9903604; doi:10.1186/s12870-023-04078-1)
Supplement: Supplementary file 1 — Additional file 1: Fig. S1. Principal component analysis (PCA) of the RNA-seq data at six time points. Mean fragments per kilobase of transcript per million mapped read (FPKM) values of three biological replicates are used for each genotype at each time point. [file 12870_2023_4078_MOESM1_ESM.docx]

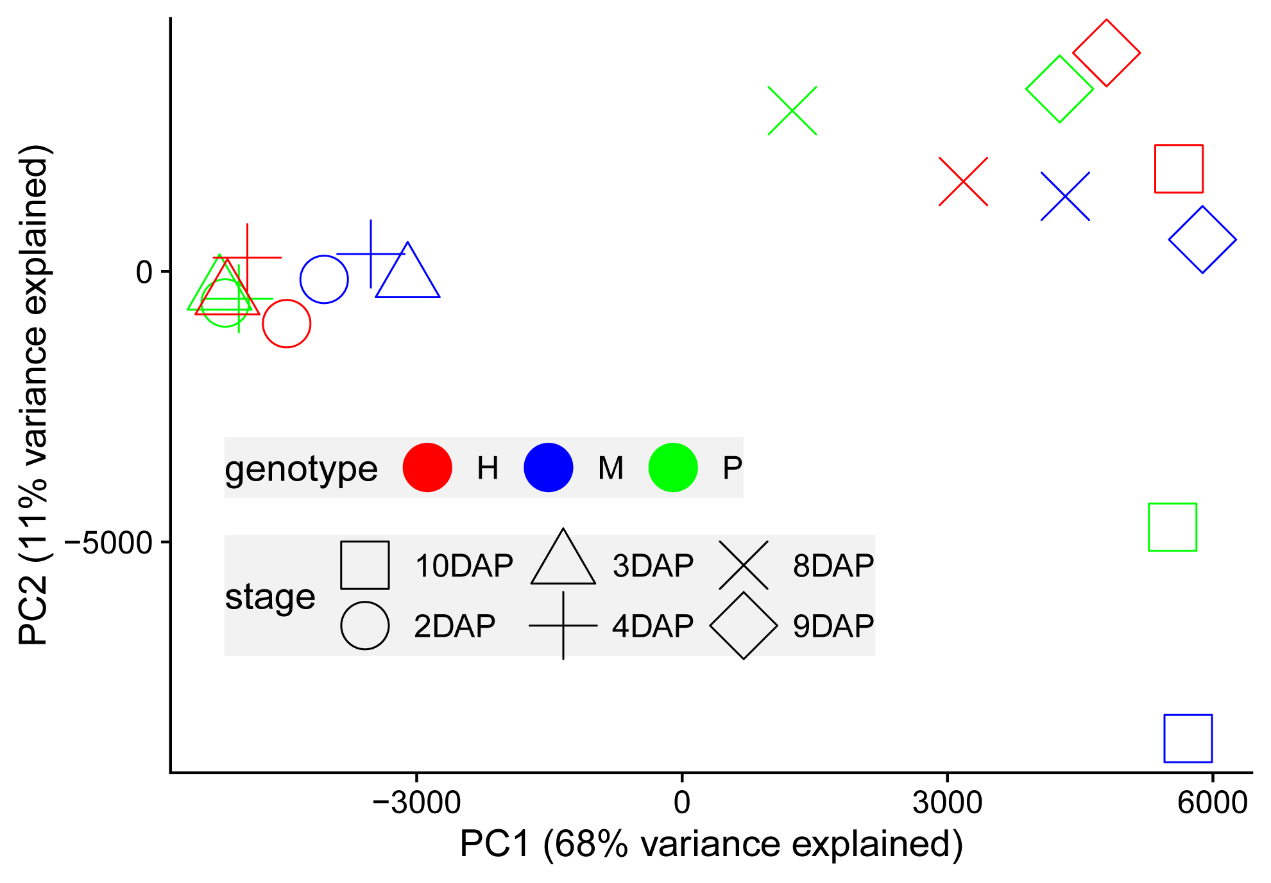


**Fig. S1** Principal component analysis (PCA) of the RNA-seq data at six time points.

Mean fragments per kilobase of transcript per million mapped read (FPKM) values of three biological replicates are used for each genotype at each time point.
