# Supplementary figures and images for: Dynamic patterns of gene expression and regulatory variation in the maize seed coat
Source: BMC Plant Biol. 2023 Feb 7;23:82. doi: 10.1186/s12870-023-04078-1 (PMC9903604; doi:10.1186/s12870-023-04078-1)

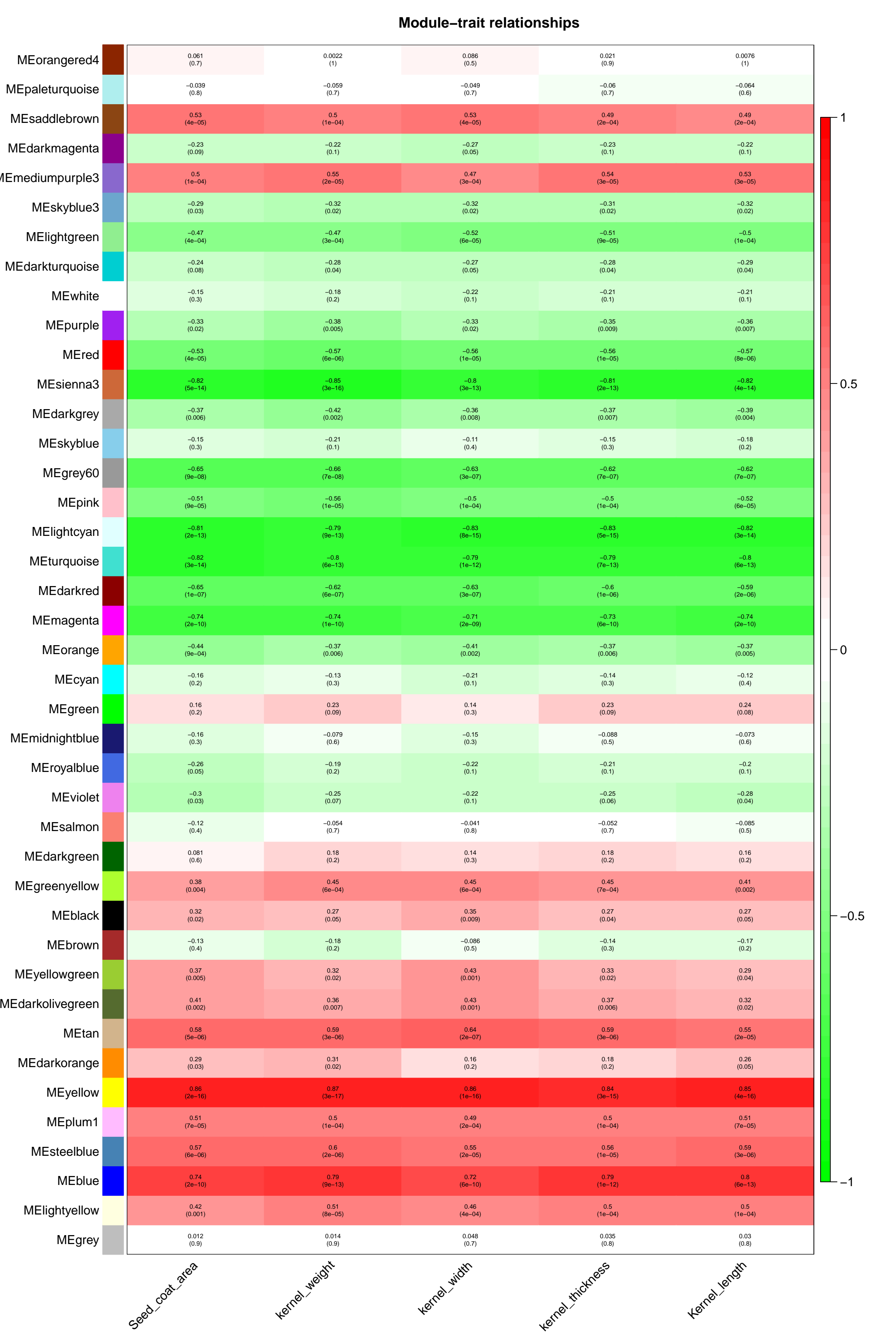

Supplement: Supplementary file 2 — Additional file 2: Fig. S2. Module-trait relationship of the module eigengene correlation with seed coat area, kernel weight, kernel width, kennel thickness and kernel length using WGCNA. [file 12870_2023_4078_MOESM2_ESM.pdf]
