# Supplementary material for: Dynamic patterns of gene expression and regulatory variation in the maize seed coat
Source: BMC Plant Biol. 2023 Feb 7;23:82. doi: 10.1186/s12870-023-04078-1 (PMC9903604; doi:10.1186/s12870-023-04078-1)
Supplement: Supplementary file 3 — Additional file 3: Fig. S3. Gene networks of hub genes with MEsienna3 module. Top 100 of genes with weight value are shown according their weight value with these hub genes. Genes with a degree value ≥ 10 were marked red. [file 12870_2023_4078_MOESM3_ESM.docx]

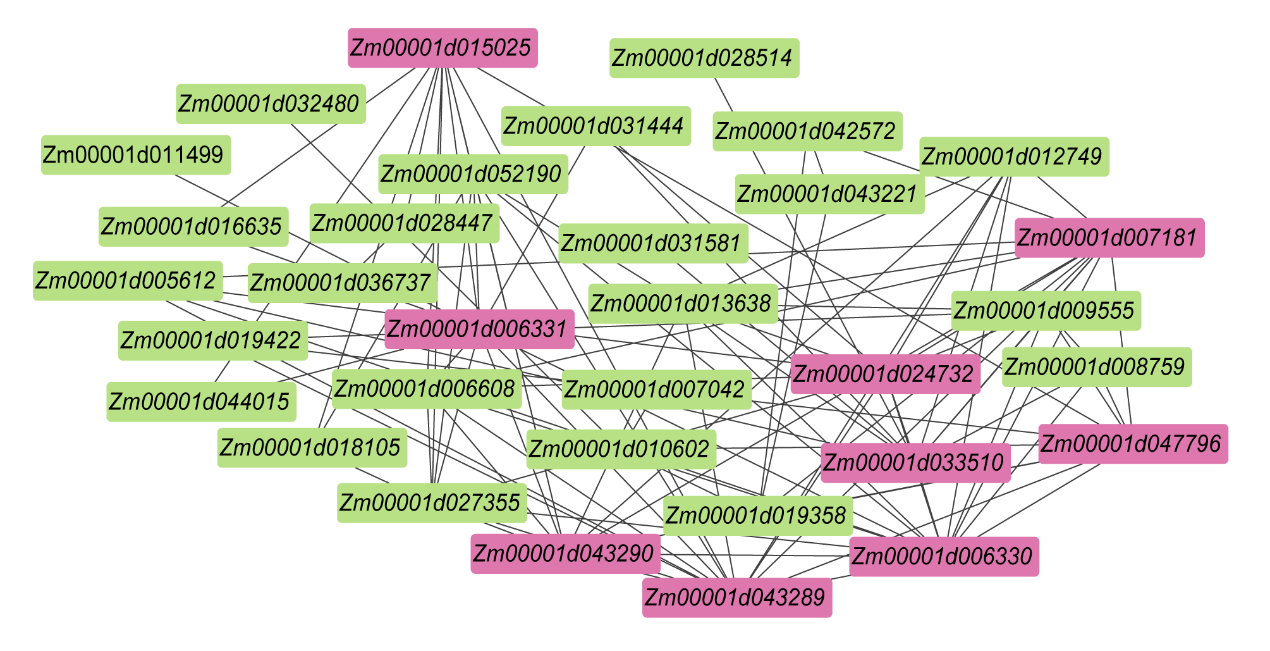


**Fig. S3** Gene networks of hub genes with MEsienna3 module.

Top 100 of genes with weight value are shown according their weight value with these hub genes. Genes with a degree value ≥ 10 were marked red.
