# Supplementary material for: Dynamic patterns of gene expression and regulatory variation in the maize seed coat
Source: BMC Plant Biol. 2023 Feb 7;23:82. doi: 10.1186/s12870-023-04078-1 (PMC9903604; doi:10.1186/s12870-023-04078-1)
Supplement: Supplementary file 4 — Additional file 4: Fig. S4. The relative expression levels of 9 differentially expressed genes on 3DAP as assessed by qRT-PCR. [file 12870_2023_4078_MOESM4_ESM.docx]

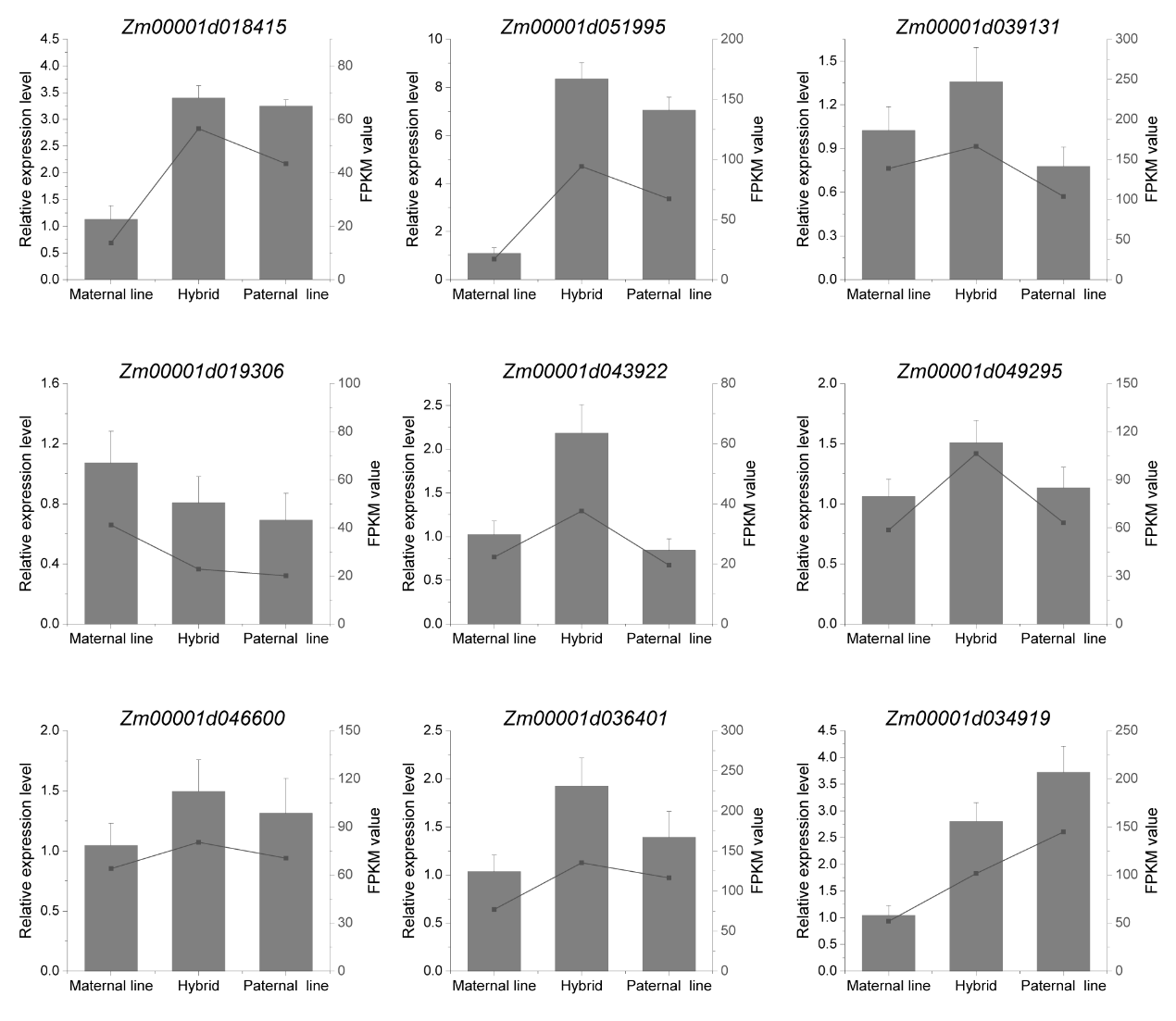


**Fig. S4** The relative expression levels of 9 differentially expressed genes on 3DAP as assessed by qRT-PCR.
